# Effects of calcium on the incidence of recurrent colorectal adenomas: A systematic review with meta-analysis and trial sequential analysis of randomized controlled trials

**DOI:** 10.1097/MD.0000000000007661

**Published:** 2017-08-11

**Authors:** Sajesh K. Veettil, Siew Mooi Ching, Kean Ghee Lim, Surasak Saokaew, Pochamana Phisalprapa, Nathorn Chaiyakunapruk

**Affiliations:** a School of Pharmacy/School of Postgraduate Studies, International Medical University, Kuala Lumpur; b Department of Family Medicine, Faculty of Medicine and Health Sciences, Universiti Putra Malaysia, Serdang, Malaysia; c Malaysian Research Institute on Ageing, Universiti Putra Malaysia, Serdang, Malaysia; d Clinical School, Department of Surgery, International Medical University, Seremban, Negeri Sembilan, Malaysia; e Center of Health Outcomes Research and Therapeutic Safety (Cohorts), School of Pharmaceutical Sciences, University of Phayao, Thailand; f School of Pharmacy, Monash University Malaysia, Bandar Sunway, Selangor, Malaysia; g Center of Pharmaceutical Outcomes Research, Department of Pharmacy Practice, Faculty of Pharmaceutical Sciences, Naresuan University, Phitsanulok; h Unit of Excellence on Herbal Medicine, School of Pharmaceutical Sciences, University of Phayao; i Division of Ambulatory Medicine, Department of Medicine, Faculty of Medicine Siriraj Hospital, Mahidol University, Bangkok, Thailand; j School of Pharmacy, University of Wisconsin, Madison, USA; k Asian Centre for Evidence Synthesis in Population, Implementation and Clinical Outcomes (PICO), Health and Well-being Cluster, Global Asia in the 21st Century (GA21) Platform, Monash University Malaysia, Bandar Sunway, Selangor, Malaysia.

**Keywords:** calcium, chemoprevention, colorectal adenomas, meta-analysis, randomized controlled trials, systematic review, trial sequential analysis

## Abstract

**Background:**

Protective effects of calcium supplementation against colorectal adenomas have been documented in systematic reviews; however, the results have not been conclusive. Our objective was to update and systematically evaluate the evidence for calcium supplementation taking into consideration the risks of systematic and random error and to GRADE the evidence.

**Methods:**

The study comprised a systematic review with meta-analysis and trial sequential analysis (TSA) of randomized controlled trials (RCTs). We searched for RCTs published up until September 2016. Retrieved trials were evaluated using risk of bias. Primary outcome measures were the incidences of any recurrent adenomas and of advanced adenomas. Meta-analytic estimates were calculated with the random-effects model and random errors were evaluated with trial sequential analyses (TSAs).

**Results:**

Five randomized trials (2234 patients with a history of adenomas) were included. Two of the 5 trials showed either unclear or high risks of bias in most criteria. Meta-analysis of good quality RCTs suggest a moderate protective effect of calcium supplementation on recurrence of adenomas (relative risk [RR], 0.88 [95% CI 0.79–0.99]); however, its effects on advanced adenomas did not show statistical significance (RR, 1.02 [95% CI 0.67–1.55]). Subgroup analyses demonstrated a greater protective effect on recurrence of adenomas with elemental calcium dose ≥1600 mg/day (RR, 0.74 [95% CI 0.56–0.97]) compared to ≤1200 mg/day (RR, 0.84 [95% CI 0.73–0.97]). No major serious adverse events were associated with the use of calcium, but there was an increase in the incidence of hypercalcemia (*P* = .0095). TSA indicated a lack of firm evidence for a beneficial effect. Concerns with directness and imprecision rated down the quality of the evidence to “low.”

**Conclusion:**

The available good quality RCTs suggests a possible beneficial effect of calcium supplementation on the recurrence of adenomas; however, TSA indicated that the accumulated evidence is still inconclusive. Using GRADE-methodology, we conclude that the quality of evidence is low. Large well-designed randomized trials with low risk of bias are needed.

## Abstract 2

Supplemental Digital Content is available in the text

## 1 Introduction

Colorectal cancer (CRC) is the third most commonly diagnosed cancer and is the fourth leading cause of cancer death worldwide.^[1]^ Although several screening strategies are available for detection and removal of asymptomatic adenomas and finding the early stages of colorectal cancer, their acceptance continues to be low.^[2]^ Moreover, even after removal of adenomas, the recurrence rate is reasonably high.^[3–5]^ Therefore, there is increased focus on the potential use of chemo-preventive agents to reduce the incidence of recurrent colorectal adenomas and colorectal cancer.

The effects of several drugs and micronutrients for the prevention of colorectal cancer and its precursor (adenomas), or both, in populations at different risks have been investigated in several epidemiologic studies including randomized controlled trials (RCTs).^[6–8]^ The results of previous meta-analyses regarding the association between calcium intake and colorectal adenoma risk have not always been consistent.^[8–12]^ Four previous meta-analyses of RCTs ^[8–11]^ that have examined the effect of supplemental calcium compared with placebo on adenoma recurrence demonstrated moderate to larger protective effects (20–26% relative risk reduction). However, the latest one^[12]^ does not demonstrate a greater protective effect (only 11–13% relative risk reduction) for calcium.

Meta-analyses including the latest one, merely considered some bias components,^[10]^ included trials with high or unclear risk of bias in the meta-analyses^[10,12]^ and did not GRADE the evidence.^[9–11]^ It is recommended that review authors do not combine studies at different risk of bias in analyses. When risks of bias vary across studies in a meta-analysis, the major approach to incorporating risk of bias assessments is to restrict meta-analyses to studies at low (or lower) risk of bias or to stratify studies according to the risk of bias.^[13,14]^ Since the overall beneficial results of the meta-analyses were unduly influenced by low-quality studies, it is important to consider any of these approaches to ease the decision making in an analysis.

Moreover, the latest meta-analysis^[12]^ used all subjects from a recent randomized controlled trial (RCT) ^[15]^ who received calcium and placebo with or without vitamin D, rather than the number of subjects who received calcium alone and placebo in the analysis. Since the data on the number of subjects who received calcium alone and placebo were available from the recent RCT, the use of these data in a meta-analysis which compares calcium against placebo appear to be more meaningful.^[16]^ Furthermore, the risk of type-I errors has not previously been assessed in this field, but growing evidence suggests this as one of the major problems of spurious findings in a meta-analysis comprising a small number of RCTs and patients. Some “positive” meta-analytic results may be due to the play of chance (random error) rather than due to some underlying “true” intervention effect.^[17,18]^ Trial sequential analysis (TSA) considers the risks of random errors and provides the necessary sample size for the meta-analysis and boundaries that determine whether the evidence in a meta-analysis is conclusive.^[18]^

For these reasons, the retrieved outcomes from previous meta-analyses may not justify the conclusion. Therefore, we carried out an updated systematic review with meta-analysis of RCTs concerning the clinical effectiveness of calcium supplementation compared with placebo in reducing the recurrence of colorectal adenomas in subjects with history of colorectal adenomas, taking into account the risks of systematic errors (bias) and random errors (play of chance). To quantify the estimated effect of calcium, we conducted meta-analyses^[14]^ and trial sequential analyses (TSAs).^[18]^ We also summarized the evidence using GRADE.^[19]^

## 2 Methods

### 2.1 Design and data sources

This study was conducted as a part of a systematic review and network meta-analysis of chemopreventive interventions for colorectal cancer which has been registered (registration number: CRD42015025849) with the PROSPERO (International Prospective Register of Systematic Reviews), previously. A complete description of the parent study design and methods has been published elsewhere.^[20]^ We followed the Cochrane Handbook for Systematic Reviews of Interventions for the planning and conduct of this meta-analysis.^[14]^ The reporting followed the Preferred Reporting Items for Systematic reviews and Meta-Analyses (PRISMA) guidelines.^[21]^

We identified relevant studies by a systematic search of MEDLINE 2008 to September 2016 (Via Ovid), MEDLINE In-Process & Other Non-Indexed Citations (Via Ovid), Embase 2008 to September 2016 (Via Ovid), Cochrane CENTRAL Register of Controlled Trials (September 2016, Via Ovid), CINAHL plus (January 2008 to September 2016), International Pharmaceutical Abstracts (September 2016), and clinicaltrials.gov website (September 2016). We developed the search strategy in MEDLINE and modified it for other databases (Supplemental Table 1). Search was restricted to studies published from 2008 onwards because studies published up to 2007 could be identified from the previous reviews.^[8,10,11]^ To identify studies not captured by database searches, we manually checked the reference lists of published systematic reviews and identified articles.

Studies included were RCTs that met the following criteria: participants were adults with history of colorectal cancer or adenomas; interventions were supplemental calcium at any dose for at least 1 year; comparators were placebo or no treatment; and primary outcomes were the incidences of any recurrent adenomas and of advanced adenomas. We excluded RCTs reported the efficacy of combination of supplemental calcium with other chemopreventive agents with evidence of efficacy against recurrent colorectal adenomas and trials in adults with history of familial cancer syndromes (such as the Lynch syndrome).

### 2.2 Data extraction and quality assessment

Requisite data were extracted independently and in duplicate by 2 reviewers into a data extraction form (SKV, SMC). Two reviewers (SKV, SMC) independently assessed the risk of bias within each study by using a Cochrane risk of bias instrument.^[13,14]^ We evaluated sequence generation, allocation concealment, blinding of participants and personnel, blinding of outcome assessment, incomplete outcome data, selective outcome reporting, and other sources of bias. Reviewers resolved disagreements by discussion, and 1 of 2 arbitrators adjudicated any unsolved disagreements. When risks of bias vary across included studies, we stratified studies according to the risk of bias and performed the sensitivity analyses separately for low-bias risk trials, high or unclear risks of bias trials, and all trials.^[13,14]^

### 2.3 Statistical analysis

All meta-analyses were performed using a random-effects model to estimate the effect size such as the pooled relative risk (RR) and 95% confidence intervals (CI) incorporating within and between-study heterogeneity. If unsuitable due to the heterogeneity and/or small number of studies, a narrative overview of the findings of included studies were presented with tabular summaries of extracted data. Heterogeneity between trials was assessed by considering the *I*^2^ statistic alongside the Chi^2^. An *I*^2^ estimate greater than or equal to 50%, accompanied by statistical significant Chi statistic, was interpreted as evidence of a substantial levels of heterogeneity.^[14]^ Analyses were performed using STATA 14.1 software. We assessed publication bias using funnel plot asymmetry testing and Egger's regression test.^[22]^

Meta-analyses might result in type-I errors owing to an increased risk of random error when smaller numbers of RCTs and patients are involved, and due to repeated significance testing when a cumulative meta-analysis is updated with new trials.^[17,18]^ Therefore, to avoid random errors, we performed trial sequential analyses using TSA software package (available at http://www.ctu.dk),^[23]^ which combines information size estimation for meta-analysis (cumulated sample size of included trials) with an adjusted threshold for statistical significance in the cumulative meta-analysis. TSA provides the necessary sample size for our meta-analysis and boundaries that determine whether the evidence in our meta-analysis is reliable and conclusive.^[18]^ Where the study not reported the actual event data, or if we observed a meta-analysis with substantial levels of heterogeneity, we avoided performing TSA.

The Grading of Recommendations, Assessment, Development and Evaluation (GRADE) approach was used to rate the quality of evidence of estimates (high, moderate, low, and very low) derived from meta-analyses using the GRADEpro GDT software. Reviewers independently assessed the confidence in effect estimates for all outcomes using the following categories: risk of bias, inconsistency, indirectness, imprecision, and publication bias (Supplemental Table 2).^[24,25]^

## 3 Results

### 3.1 Description of included trials

Five RCTs^[15,26–29]^ comparing supplemental calcium versus placebo for the prevention of recurrent colorectal adenomas in increased-risk population (subjects with a previous history of colorectal cancer or adenomas) met the eligibility criteria and were included. Another one RCT^[30]^ was identified for supplemental calcium, but did not meet the eligibility criteria, and was excluded with reason (Supplemental Table 3). Supplemental Figure 1 shows the search process. Table 1 describes the characteristics of included studies. A total of 2234 participants who completed the follow-up colonoscopy in the 5 trials were included in the meta-analysis. All 5 trials included both men and women with a history of adenomas. The length of follow-up from recruitment to the study was 3 years in 2 trials,^[27,28]^ 4 years in 1 trial,^[26]^ and 5 years in the remaining trials.^[15,29]^ All trials employed comparisons of calcium against placebo, except the Hofstad study,^[28]^ that examined mixed intervention consisting of calcium and antioxidants against placebo. Since antioxidants were without any significant effects on adenoma recurrence as described by previous reviews,^[8,31]^ the results from the Hofstad study may represent the effect of calcium alone on adenomas; hence, we included this study in our review. The dose per day of elemental calcium^[32]^ used in 3 trials ranged from 720^[29]^ to 1200 mg^[15,26]^ and in remaining trials it was to 1600^[28]^ to 2000 mg^[27]^. All 5 trials used an adenoma endpoint. In all trials, compliance with the study treatments was generally good, with a mean pill-taking rate in the approximate range 69% to 80%.

**Table 1:**
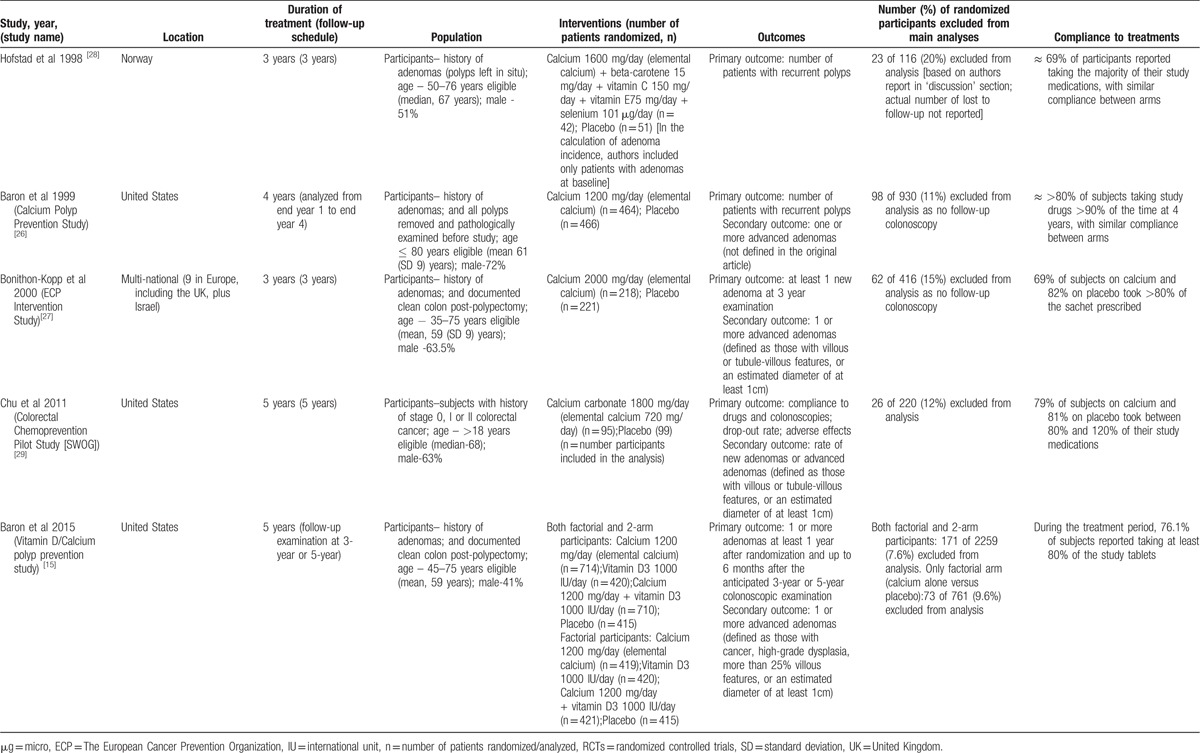
Characteristics of RCTs comparing supplemental calcium versus placebo for effectiveness in reducing the recurrence of colorectal adenomas.

### 3.2 Quality assessment of the trials

**Figure 1:**
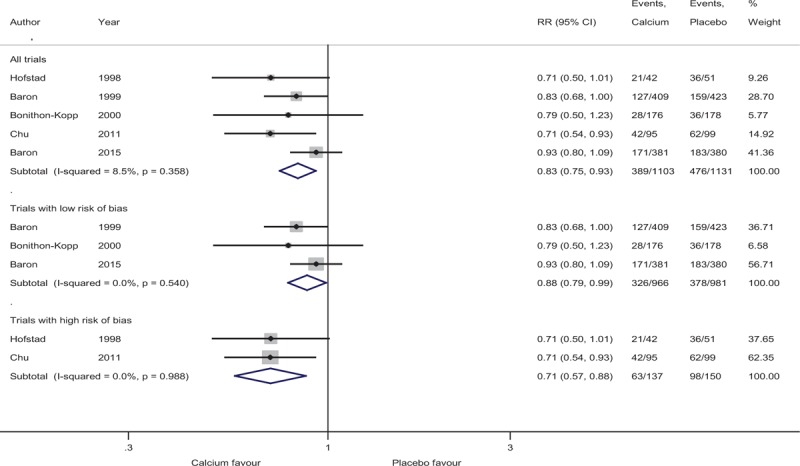
Incidence of recurrent adenomas in subjects with a history of adenomas randomized to calcium.

The risk of bias table for all trials and risk of bias graph are illustrated in Supplemental Table 4 and Supplemental Figure 2. Among 5 RCTs, 3^[15,26,27]^ had low risk of bias in all criteria and the remaining 2 trials^[28,29]^ showed either unclear or high risks of bias in most criteria. Among these 2 RCTs^[28,29]^, allocation concealment was probably not done in the SWOG Calcium Chemoprevention Pilot Study^[29]^ and was unclear in Hofstad study.^[28]^ The methods of randomization and blinding were inadequate in both trials. Moreover, the control event rate was considerably high in these 2 trials compared to large, high-quality RCTs (refer Fig. 1). In all 5 trials, between 9.6% and 20% of randomized participants were excluded from analysis and were balanced in numbers and reasons across intervention groups.

Because of differences in risk of bias, we present the effect estimates of calcium in the trials with low risk of bias, high or unclear risks of bias separately, but also a meta-analysis of all trials using a random-effects model.

### 3.3 Recurrence of any adenomas

Figure 1 summarizes the random-effects meta-analysis comparing supplemental calcium to placebo. Among 2234 participants for whom follow-up colonoscopy results were available from all 5 RCTs, adenomas of any type were found in 865 (38.7%) participants. We used the event rates from the subjects (factorial arm participants) who received calcium supplement alone and placebo from the Baron et al (2015) study^[15]^ in our meta-analysis (the data were provided by the author on request).

Quantitative pooling of results from all RCTs indicated that the use of supplemental calcium lasting 3 to 5 years showed a statistically significant 17% reduction in risk of any recurrent adenomas (RR, 0.83 [95% CI 0.75–0.93]), with low heterogeneity between the studies (*I*^2^ = 8.5%, *P* = .36).

### 3.4 Sensitivity analyses based on bias risk of the trials

In the sensitivity analysis of 3 trials with low bias risk (Fig. 1), we found a moderate 12% reduction in the recurrence of any adenomas (RR, 0.88 [95% CI 0.79–0.99]) in patients who were administered supplemental calcium versus placebo, with no heterogeneity (*I*^2^ = 0%, *P* = .54). However, a greater reduction of 29% (RR, 0.71 [95% CI 0.57–0.88]) was observed in subgroup analysis of 2 trials with high bias risk, with no heterogeneity (*I*^2^ = 0%, *P* = .99) (Fig. 1).

### 3.5 Recurrence of advanced adenomas

**Figure 2:**
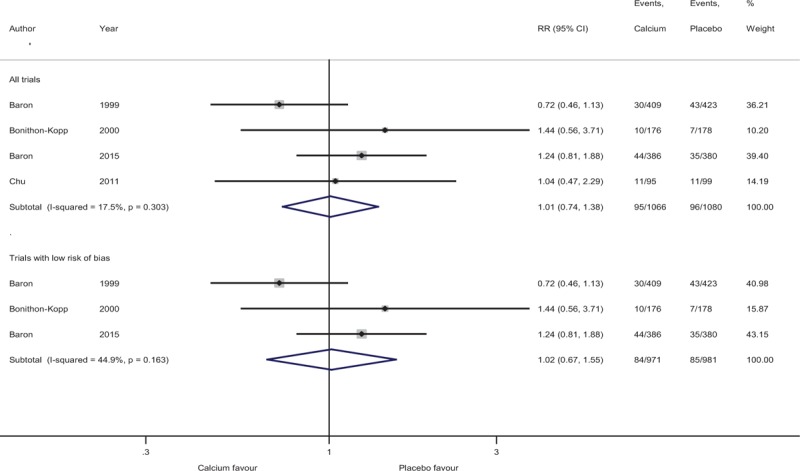
Incidence of recurrent advanced adenomas in subjects with a history of adenomas randomized to calcium.

Figure 2 summarizes the random-effects meta-analysis comparing supplemental calcium versus placebo on advanced adenomas. None of the trials reported a statistically significant beneficial effect for calcium on recurrence of advanced adenomas. Their overall occurrence in all 4 RCTs was 8.9% in calcium groups and 8.8% in placebo groups.

In meta-analysis, the association between supplemental calcium and recurrence of advanced adenomas in trials with low risk of bias (RR, 1.02 [95% CI 0.67–1.55]) and using all trials (RR, 1.01 [95% CI 0.74–1.38) did not reach statistical significance. There was low (*I*^2^ = 17.5%) to moderate level (*I*^2^ = 44.9%) of heterogeneity observed for both analyses.

### 3.6 Subgroup analyses

In the subgroup analysis (Supplemental Figure 3) of 3 trials with elemental calcium dose ≤ 1200 mg/day, we found a 16% reduction in the recurrent of any adenomas (RR, 0.84 [95% CI 0.73–0.97]), with a moderate level of heterogeneity (*I*^2^ = 38.5%, *P* = .19). However, a greater reduction of 26% (RR, 0.74 [95% CI 0.56–0.97]) was observed in the subgroup analysis of 2 trials with elemental calcium dose ≥ 1600 mg/day, with no heterogeneity (*I*^2^ = 0%, *P* = .70). Subgroup analyses demonstrated no statistically significant association with the reduction of advanced adenomas in any doses (Supplemental Figure 4).

### 3.7 Trial sequential analysis

**Figure 3:**
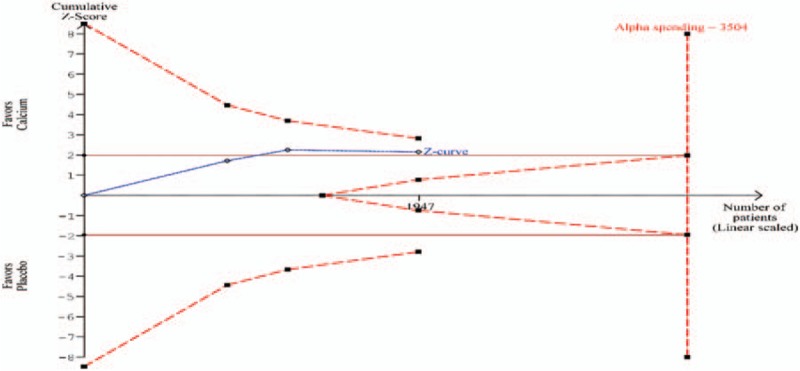
Trial sequential analysis (TSA) assessing the effect of supplemental calcium on recurrent adenoma incidence. The information size required to demonstrate or reject a 12% relative reduction (low-bias risk trail estimate) based on an assumption of 38.5% of control group event proportion (median proportion of incidence of recurrent adenomas in the control group) with type 1 error of 5% two-sided and type II error of 20% is 3504 patients. The cumulated Z-curve (blue) crosses the traditional boundary but not the trial sequential monitoring boundary indicating the lack of firm evidence for a beneficial effect of 12% relative risk reduction of the intervention when the analysis is adjusted for repetitive testing on accumulating data. There is insufficient information to reject or detect the anticipated intervention effect (12%) as the required information size is not yet reached. TSA = trial sequential analysis.

For supplemental calcium, TSA for any recurrent adenomas based on the information size adjusting for the presence of heterogeneity among 3 trials with low bias risk is shown in Fig. 3. The required heterogeneity-adjusted information size to demonstrate or reject a 12% relative risk reduction of recurrence of any adenomas based on the trials with a low risk of bias using a control event proportion of 38.5%, an alpha (type-1 error) of 5% two-sided and a beta of 20% (power = 80%) is 3504 patients. The number of patients included in the meta-analysis did not exceed the required information size and alpha-spending monitoring boundary was not reached or crossed, indicating that the cumulative evidence is inconclusive for 12% relative risk reduction of any recurrent adenomas.

We did not perform TSA for supplemental calcium on the incidence of recurrent advanced adenomas due to the non-significant effect and substantial heterogeneity identified during meta-analysis.

### 3.8 Adverse effects

The included studies reported data on constipation, diarrhea, hypercalcemia, cardiovascular adverse events, hypercreatininemia and urolithiasis (Supplemental Table 5). The incidence of hypercalcemia was statistically significantly higher in the calcium group than the control group (*P* = .0095). Calcium supplementation was associated with significantly fewer myocardial infarctions than participants who were assigned to no calcium supplementation (*P* = .0375) in 1 trial.^[15]^ There were no statistically significant differences between groups in terms of other adverse effects.

### 3.9 GRADE Summary of evidence for calcium

Randomized trials without important limitations are rated high on the GRADE scale. Since we have included only trials with low bias risk to GRADE the summary of evidence, there was no serious risk of bias in the trials. There was no serious inconsistency identified between trials. Interventions were delivered in different doses and the duration of follow-up varied among these studies *(*refer Table 1*)*. Hence, we downgraded the rating because of questionable directness in the summary. In context with the evidence from TSA, the optimal information size criterion is not met *(*refer Fig. 3*)*; hence, we chose to downgrade on imprecision.

Our application of GRADE-methodology led us to conclude that the accumulated evidence for calcium supplementation is of low quality for adenoma prevention. A summary of findings and strength of evidence is shown in (Table 2).

**Table 2:**
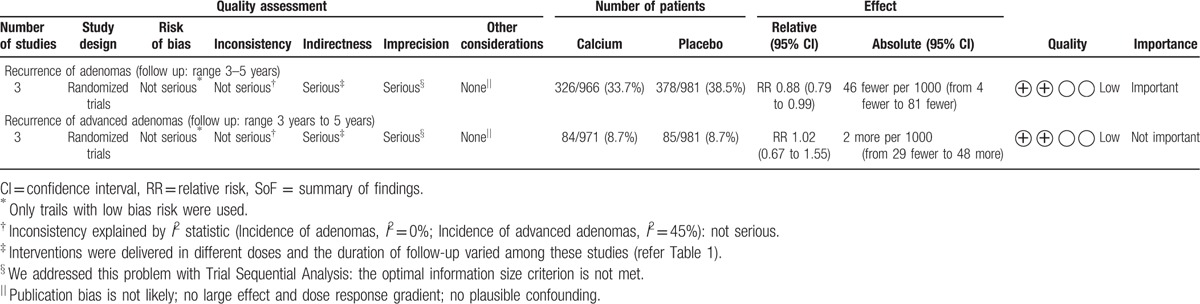
Summary of findings (SoF).

### 3.10 Publication bias

Based on visual inspection of the funnel plots as well as on quantitative measurement that used the Egger regression test, there was weak evidence of publication bias (Supplemental Figures 5 and 6).^[14,33]^

## 4 Discussion

We have identified 5 previous systematic reviews with meta-analyses^[8–12]^ of RCTs examining the effects of calcium supplementation on colorectal adenoma prevention. The effects of calcium supplementation on adenoma recurrence from these studies were not always consistent. Using 2 similar good quality trials^[26,27]^ in the meta-analyses, a review by Cooper et al^[8]^ found a significant 18% risk reduction of any recurrent adenomas; however, a greater protective effect of 26% was reported in Weingarten et al^[11]^ review. Although both meta-analyses used the same trials, the possible explanation for this discrepancy could be the use of numbers of randomized patients as the denominator in the analysis, rather than the numbers of patients who completed the follow-up study (colonoscopy) in the Weingarten et al review. This approach assumes that none of the patients who were lost to the follow-up experienced the adenoma recurrence in the Weingarten et al review.^[34,35]^ Hence, the relative incidence of adenoma recurrence in the calcium arm of the Weingarten et al review was smaller than that reported in the original intention-to-treat analysis, and so may have contributed to the larger protective effect of calcium supplementation as seen in Weingarten et al review. In addition to the 2 trials used in the earlier reviews, Carroll et al^[9]^ and Shaukat et al^[10]^ performed a 3-trial^[15,26,28]^ meta-analyses, by including 1 more trial (the Hofstad et al study)^[28]^; they found a significant 20% risk reduction associated with calcium. On the other hand, the latest review by Bonovas et al^[12]^ using 4 trials, including the Baron et al (2015) study^[15]^ and Colorectal Chemoprevention Pilot Study,^[29]^ and excluding the Hofstad et al study,^[28]^ found only a modest protective effect of calcium supplementation in prevention of recurrent adenomas (11–13% risk reduction).

However, in our analysis, we have identified an unclear or high risk of bias for 1 or more key domains as per Cochrane risk of bias instrument in both the Hofstad et al^[28]^ study and the Colorectal Chemoprevention Pilot Study.^[29]^ Since the overall beneficial results of the meta-analysis were unduly influenced by these studies, we graded these studies as trials with high bias risk in our meta-analysis and analyzed these separately.^[13,14]^ Moreover, the review by Bonovas et al have used event data of all the subjects who received calcium and placebo with or without vitamin D as reported in the Baron et al^[15]^ (2015) study, rather than the number of subjects who received calcium alone and placebo in their meta-analysis. Since the data were provided by the author on request, we used the event rates from the subjects who received calcium alone and placebo from the Baron et al^[15]^ (2015) study in our meta-analysis. Moreover, the risk of type-I errors has not previously been assessed in this field, but growing evidence suggests this as one of the major problems of spurious findings due to meta-analyses ^[17,36]^ and may therefore provide a valuable addition.

For these reasons, we carried out an updated systematic review with meta-analysis taking into account the risks of bias, evaluated random errors and incorporated the GRADE rating, thus broadening the base for a well-founded judgment of the evidence. Among 5 RCTs^[15,26–29]^ identified for calcium supplementation to describe the effects on recurrent adenoma incidence, 3^[15,26,27]^ were of good quality, with high compliance and generally with high follow-up rates; however, others^[28,29]^ were associated with substantial risk of bias. The updated summary of the effects of calcium from the high-quality trials suggests that the regular use of calcium supplementation lasting 3 to 5 years seems to reduce the incidence of recurrent colorectal adenomas, with a pooled 12% relative risk reduction in patients with a previous history of colorectal adenomas. However, the association between calcium supplementation and advanced adenomas did not reach statistical significance. This is similar to the results reported by a recent systematic review.^[12]^ However, meta-analyses of all trials^[15,26–29]^ irrespective of risks of bias and trials^[28,29]^ with high risks of bias demonstrated a more substantial protective effect of 17% and 29% relative risk reductions in the recurrence of any adenomas, respectively. This is concordant to the usual observation, in which intervention effects are usually overestimated in high-bias risk trials.^[14]^

Subgroup analyses demonstrated that the risk of adenoma recurrence continued to decrease with increasing calcium intake, a finding consistent with the previously published dose–response meta-analysis of prospective observational studies.^[37]^ Though we observed no serious adverse events with the use of calcium (elemental calcium dose ranged from 720 to 2000 mg/day) lasting 3 to 5 years in patients with a previous history of adenomas, more high quality evidence has shown that calcium supplements (elemental calcium ≥500 mg/day) can increase the risk of cardiovascular events,^[38,39]^ especially myocardial infarction (an increased risk of about 30%).^[38]^ Although calcium supplements modestly increase bone density^[40]^ and have a marginal efficacy against fracture,^[41,42]^ the risk of cardiovascular events suggests that a reassessment of the role of calcium (elemental calcium <500 mg/day) as a chemopreventive agent is warranted.

The present meta-analysis, based on the low-bias risk trials, comprises only a few RCTs and did not include a substantial number of patients. Therefore, the modest chemopreventive effect of calcium supplements against colorectal adenomas seen in our analysis could be because of random errors. TSA showed that there is lack of firm evidence for a beneficial effect and an insufficient information size to accept the anticipated intervention effect. Thus, the question whether calcium is beneficial for adenoma recurrence prevention remains unanswered. Using GRADE methodology, we are led to conclude that the quality of the evidence is low.

There are some limitations to this systematic review. The number of available high-quality trials was limited. RCTs included in this review of calcium were similar but not identical with regard to follow-up and the dose. Because the follow-up of studies was not sufficiently long, we could not explore the long-term effects of calcium supplementation on the recurrence of adenomas and the progression to cancer. TSA demonstrated that the number of patients included in the meta-analysis did not exceed the required information size and we have no conclusive evidence in favor of calcium supplementation on adenoma recurrence.

In summary, the available good quality randomized trials suggest a possible beneficial effect of calcium supplementation on recurrence of adenomas without significant important adverse effects, but accumulated evidence is still inconclusive based on low-bias risk trials. Using GRADE methodology, we conclude that the quality of the evidence is low. However, the absence of good quality evidence is not always evidence of absence of an effect. Large, high quality randomized trials comparing calcium versus placebo are still needed. Moreover, cardiovascular adverse effects associated with calcium supplementation in the light of new evidence suggest that the benefit of calcium chemoprevention would need to be carefully weighed against its harms.

## Acknowledgments

The authors wish to thank Professor Brian L Furman, Strathclyde Institute of Pharmacy and Biomedical Sciences, Glasgow, UK, for his valuable comments and support which helped to improve the manuscript. The authors wish to thank Prof Dato’ Dr (Mrs) Kew Siang Tong, School of Medicine, International Medical University and Dr. Muhammad Radzi bin Abu Hassan, Head of Gastroenterology Service Ministry of Health, Malaysia, for their expertise and advice during the development of this protocol. The authors also wish to thank Mr. Razman Shah Mohd Razali, reference librarian, International Medical University for providing the full text articles whenever needed.

## Supplementary Material

Supplemental Digital Content
