# Supplementary material for: Effects of calcium on the incidence of recurrent colorectal adenomas: A systematic review with meta-analysis and trial sequential analysis of randomized controlled trials
Source: Medicine (Baltimore). 2017 Aug 11;96(32):e7661. doi: 10.1097/MD.0000000000007661 (PMC5556213; doi:10.1097/MD.0000000000007661)
Supplement: Supplemental Digital Content [file medi-96-e7661-s001.docx]

**Supplemental Figure 1:** A flow of study selection

Records excluded
(n =3840)

Full-text articles assessed for eligibility
(n = 145)

Records screened
(n =3985)

Records after duplicates removed
(n =3985)

Additional records identified through other sources
(n = 120)

Records identified through database searching
(n = 4989)

Identification

Full-text articles excluded, with reasons (As per parent study protocol) (n = 70)

Not an eligible study design (n=26)

Not an eligible population (n=2)

Not an eligible intervention (n=7)

Not an eligible outcome (n=32)

Duplicate (n=3)

Studies identified (RCTs, n = 6); 1 RCT excluded with reason

Included

Eligibility

Screening

Studies included in the systematic review of all interventions (Parent study)
(n = 75)

After the initial screening (the title and abstract screening) of the candidate studies, we excluded 3840 studies that not met our inclusion criteria

Studies identified (RCTs, n = 6); 1 RCT excluded with reason

Included

Eligibility

Studies included in the systematic review of all interventions (Parent study)
(n = 75)

**Supplemental Figure 2:** Summary of risk of bias (ROB) of all included studies on calcium.


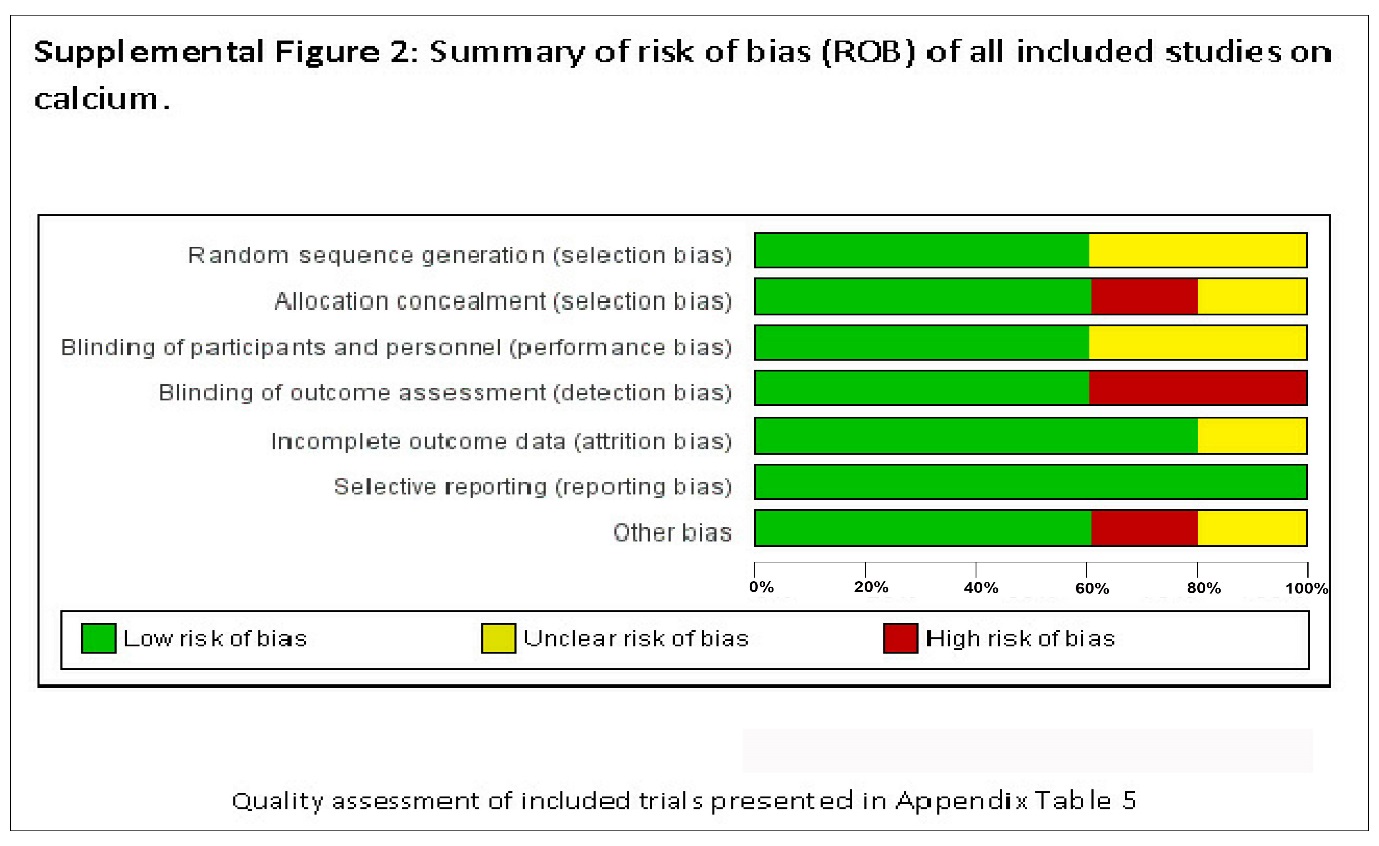


Quality assessment of included trials presented in Appendix Table 5

**Supplemental Figure 3: Subgroup analyses based on dose: incidence of recurrent adenomas**

RR=relative risk; CI: Confidence interval

**Supplemental Figure 4: Subgroup analyses based on dose: incidence of recurrent advanced adenomas**

RR=relative risk; CI: Confidence interval

**Supplemental Figure 5: Publication bias – (A) Funnel plot (B) Egger regression test for all**

**included studies of calcium on recurrent adenoma incidence**

(A)

RR=relative risk; se: standard error

(B)

Funnel plot looked somewhat asymmetric. However, Egger test demonstrated that the estimated bias coefficient is -3.007 with a standard error of 1.041, giving a p-value of 0.063. The test thus provides weak evidence for the presence of small-study effects (cut-off p-value 0.05).^1^

Reference:

Jonathan A. C. Sterne. *Meta-Analysis in Stata: An Updated Collection from the Stata Journal*. First edition. A Stata Press Publication, StataCorp LP College Station, Texas; 2009.

**Supplemental Figure 6: Publication bias – (A) Funnel plot (B) Egger regression test for all**

**included studies of calcium on recurrent advanced adenoma incidence**

(A)

RR=relative risk; se: standard error

(B)

Funnel plot looked somewhat asymmetric. However, Egger test demonstrated that the estimated bias coefficient is 0.331 with a standard error of 3.622, giving a p-value of 0.936. The test thus provides weak evidence for the presence of small-study effects (cut-off p-value 0.05).^1^

Reference:

Jonathan A. C. Sterne. *Meta-Analysis in Stata: An Updated Collection from the Stata Journal*. First edition. A Stata Press Publication, StataCorp LP College Station, Texas; 2009.

**Supplemental Table 1:** Search strategy in MEDLINE

| **Search strategy for parent study (Medline)** | |
| --- | --- |
| 1 | Terms for colorectal cancer or adenomas  (exp Colorectal Neoplasms/ OR exp Colonic Neoplasms/ OR exp Rectal Neoplasms/ OR exp Adenomatous Polyps/ OR exp Adenocarcinoma/ OR exp Intestinal Polyps/ OR exp Colonic Polyps/) OR ((colorectal cancer$.tw OR colorectal tumo$.tw OR colorectal neoplas$.tw OR colon cancer$.tw OR colon tumo$.tw OR colon neoplas$.tw OR colonic cancer$.tw OR colonic tumo$.tw OR colonic neoplas$.tw OR rectal cancer$.tw OR rectal tumo$.tw OR rectal neoplas$.tw OR rectum cancer$.tw OR rectum tumo$.tw OR rectum neoplas$.tw OR polyp$.tw OR adenoma$.tw OR adenomatous$.tw) OR (exp Adenoma/)) |
| 2 | Terms for NSAIDs and aspirin  (exp Anti-Inflammatory Agents, Non-Steroidal/ OR exp cyclooxygenase inhibitors/ OR exp  cyclooxygenase 2 inhibitors/ OR exp Aspirin/) OR (NSAID$.tw. OR Non-steroidal anti-inflammatory$.tw. OR Nonsteroidal anti-inflammatory$.tw. OR Non-steroidal antiinflammatory$.tw. OR Nonsteroidal antiinflammatory$.tw. OR Cyclo-oxygenase inhibitor$.tw. OR Cyclooxygenase inhibitor$.tw. OR Cyclooxygenase 1 inhibitor$.tw. OR Cyclooxygenase 2 inhibitor$.tw. OR COX-2 inhibitor$.tw. OR COX-2 selective inhibitor$.tw. OR COX-1 inhibitor$.tw. OR Coxib$.tw. OR Aspirin.af. OR Acetylsalicylic acid.tw.) |
| 3 | Terms for folic acid  (exp Folic Acid/ OR folate$.tw. OR folic$.tw. OR folic Acid.af.) |
| 4 | Terms for calcium  (exp Calcium, Dietary/ OR exp Calcium/ OR calcium.tw.) |
| 5 | Terms for vitamin D  (exp Cholecalciferol/ OR exp Ergocalciferols/ OR vitamin d.tw. OR Cholecalciferol$.tw. OR Ergocalciferol$.tw.) |
| 6 | Terms for antioxidants  (exp Antioxidants/ OR anti-oxidant$.tw. OR antioxidant$.tw. OR Selenium/ OR exp Vitamin A/ OR exp Carotenoids/ OR carotenoid$.tw. OR beta-carotene.tw. OR exp Ascorbic Acid/ OR vitamin c.tw. OR exp Vitamin E/ OR exp Tocopherols/ OR Tocotrienols/ OR alpha-tocopherol$.tw. OR tocopherol$.tw. OR tocotrienol$.tw.) |
| 7 | Terms for randomized controlled trial  (randomized controlled trial.pt. OR controlled clinical trial.pt. OR exp Clinical Trial/ OR Randomized controlled trials/ OR random allocation/ OR double blind method/ OR single blind method/ OR clinical trial.pt. OR placebos/ OR placebo$.ti,ab. OR random$.tw OR blind$.ti,ab.) |
| 8 | (1 AND (2 OR 3 OR 4 OR 5 OR 6) AND 7)  Limit 8 to (humans and yr="2008 - 2015") |
| 9 | Update the search: August 2015- September 2016 |

**Supplemental Table 2:** GRADE Working Group grades of evidence*

| **Grading** | **Meaning** |
| --- | --- |
| High quality | We are very confident that the true effect lies close to that of the estimate of the effect |
| Moderate quality | We are moderately confident in the effect estimate: The true effect is likely to be close to the estimate of the effect, but there is a possibility that it is substantially different |
| Low quality | Our confidence in the effect estimate is limited: The true effect may be substantially different from the estimate of the effect |
| Very low quality | We have very little confidence in the effect estimate: The true effect is likely to be substantially different from the estimate of effect |

*The quality of evidence based on grading system can be rated down based on risk of bias (i.e., low risk, unclear, high risk), indirectness (i.e., measurement outcome such as death (direct outcome) or ALT level (indirect outcome)), imprecision (i.e., wide range of 95%CI), inconsistency (or heterogeneity) and/or publication bias, to levels of moderate, low and very low quality.

**Supplemental Table 3:** Reason for exclusion of identified studies.

| **Study** | **Reason for exclusion** |
| --- | --- |
| HC Pommergaard 2016 | Randomized, double-blind, placebo-controlled trial  Interventions and participants: mixture of 0.5 μg/day calcitriol, 75 mg/day acetylsalicylic acid, and 1250 mg/day calcium carbonate (n = 209), or placebo (n = 218)  Not separate arm for calcium. Aspirin found to be effective in reducing recurrence of adenomas; hence, the results from this study not representing calcium alone. |

Reference:

Pommergaard H-C, Burcharth J, Rosenberg J, Raskov H. Aspirin, Calcitriol, and Calcium Do Not Prevent Adenoma Recurrence in a Randomized Controlled Trial. *Gastroenterology*. 2016;150(1):114-122.

**Supplemental Table 4:** Risk of bias table for all trials

| **Study** | **Random sequence generation** | **Allocation concealment** | **Blinding of participants and personnel** | **Blinding of outcome assessment** | **Incomplete outcome data** | **Selective reporting** | **Other bias** | **Judgement** |
| --- | --- | --- | --- | --- | --- | --- | --- | --- |
| **Effects of calcium on recurrent colorectal adenomas** | | | | | | | | |
| Hofstad 1998 | **unclear** | **unclear** | **unclear** | Not stated; **high risk** | 23 of 116 (20%) excluded  from analysis;  **unclear**;  (actual number of lost to follow-up patients not reported) | The study protocol is available and all of the study’s pre-specified (primary and secondary) outcomes that are of interest in the review have been reported; **low risk** | Control event rate is too high compared to large/good quality RCTs.  **unclear** | Plausible bias that seriously weakens confidence in the results. |
| Baron 1999; Calcium Polyp Prevention Study | Computer generated; **low risk** | Central pharmacy; **low risk** | Double blind; identical-appearing placebo; **low risk** | probably done, **low risk** | 98 of 930 (11%) excluded  from analysis as no follow-up  colonoscopy; **low risk** | The study protocol is available and all of the study’s pre-specified (primary and secondary) outcomes that are of interest in the review have been reported; **low risk** | The study appears to be free of other sources of bias; **low risk** | Low risk of bias for all key domains |
| Bonithon-Kopp 2000; ECP Intervention Study | Computer generated; **low risk** | Central pharmacy; **low risk** | Double blind; **low risk** | investigators were blind to the treatment; **low risk** | 62 of 416 (15%) excluded  from analysis as no follow-up  colonoscopy; **low risk** | The study protocol is available and all of the study’s pre-specified outcomes that are of interest in the review have been reported; **low risk** | The study appears to be free of other sources of bias; **low risk** | Low risk of bias for all key domains |
| Chu 2011; Colorectal Chemoprevention Pilot Study (SWOG) | **unclear** | Not stated anywhere; probably not done; **high risk** | **unclear** | **high risk** | 26 of 220 (12%) excluded from analysis. **low risk** | All of the study’s pre-specified outcomes that are of interest in the review have been reported; **low risk** | **high risk** (used participants with history of colorectal cancer stage 0, I or II); control event rate is too high compared to large-high quality RCTs on subjects with history of only adenomas. | Plausible bias that seriously weakens confidence in the results. |
| Baron 2015; VitD/Calcium polyp prevention study | Computer generated; **low risk** | Central allocation; **low risk** | Double blind; **low risk** | probably done, **low risk** | 171 of 2259 (7.6%) excluded from analysis. **low risk** | The study protocol is available and all of the study’s pre-specified outcomes that are of interest in the review have been reported; **low risk** | The study appears to be free of other sources of bias; **low risk** | Low risk of bias for all key domains |

**Supplemental Table 5:** Adverse events in all trials (calcium versus placebo)

| Adverse events | **Hofstad 1998** | | **Bonithon-Kopp 2000** | | **Chu 2011** | | **Baron 2015** | | **P*** |
| --- | --- | --- | --- | --- | --- | --- | --- | --- | --- |
|  | Calcium 1600 mg/day plus anti-oxidants (n = 42) | Placebo (n = 51) | Calcium 2000 mg/day  (n = 176) | Placebo (n = 178) | Calcium carbonate 1800mg (n=95) | Placebo (n=99) | Calcium carbonate 1200mg (n=840 ) | No calcium (n= 835 ) |  |
| Constipation | 5 | 1 | NR | NR | 19 (grade 1 to 3) | 18 (grade 1 to 2) | NR | NR | 0.3206 |
| Diarrhoea | 5 | 7 | 6 | 3 | 3 (grade 1 to 3) | 1 (grade 3) | NR | NR | 0.5425 |
| Hypercalcemia | NR | NR | NR | NR | 2 (grade 1 and 4) | 2 (grade 1) | 17 | 5 | 0.0095 |
| Urolithiasis | NR | NR | NR | NR | NR | NR | 20 | 15 | 0.4953 |
| Hypercreatininemia | NR | NR | NR | NR | 3 (grade 1) | 6 (grade 1 and 2) | 58 | 40 | 0.1631 |
| Myocardial infarction | NR | NR | NR | NR | NR | NR | 2 | 9 | 0.0375 |
| The Baron 1999 study reported that ‘medical symptoms and complications were not associated with treatment assignment. Hence, we excluded the data from Baron 1999 study.  *****P values (two-sided) are from Fisher’s exact test comparing placebo vs calcium in any dose.  n= number of participants randomized/analyzed; NR= not reported | | | | | | | | | |
